# Spatiotemporal Differentiation of MMN From N1 Adaptation: A Human ECoG Study

**DOI:** 10.3389/fpsyt.2020.00586

**Published:** 2020-06-26

**Authors:** Megumi Takasago, Naoto Kunii, Misako Komatsu, Mariko Tada, Kenji Kirihara, Takanori Uka, Yohei Ishishita, Seijiro Shimada, Kiyoto Kasai, Nobuhito Saito

**Affiliations:** ^1^ Department of Neurosurgery, The University of Tokyo, Tokyo, Japan; ^2^ Laboratory for Molecular Analysis of Higher Brain Function, RIKEN Center for Brain Science, Wako, Japan; ^3^ Department of Neuropsychiatry, Graduate School of Medicine, The University of Tokyo, Tokyo, Japan; ^4^ The International Research Center for Neurointelligence (WPI-IRCN), The University of Tokyo Institutes for Advanced Study (UTIAS), Tokyo, Japan; ^5^ Department of Integrative Physiology, Graduate School of Medicine, University of Yamanashi, Yamanashi, Japan; ^6^ Department of Neurosurgery, Jichi Medical University, Shimotuke, Japan

**Keywords:** auditory mismatch negativity (auditory MMN), N1, electrocorticography (ECoG), event-related potential (ERP), adaptation, deviance detection

## Abstract

Auditory mismatch negativity (MMN) is an electrophysiological response to a deviation from regularity. This response is considered pivotal to understanding auditory processing, particularly in the pre-attentive phase. However, previous findings suggest that MMN is a product of N1 adaptation/enhancement, which reflects lower-order auditory processing. The separability of these two components remains unclear and is considered an important issue in the field of neuroscience. The aim of the present study was to spatiotemporally differentiate MMN from N1 adaptation using human electrocorticography (ECoG). Auditory evoked potentials under the classical oddball (OD) task as well as the many standards (MS) task were recorded in three patients with epilepsy whose lateral cortices were widely covered with high-density electrodes. Close observation identified an electrode at which N1 adaptation was temporally separated from MMN, whereas N1 adaptation was partially incorporated into MMN at other electrodes. Since N1 adaptation occurs in the N1 population, we spatially compared MMN with N1 obtained from the MS task instead of N1 adaptation. As a result, N1 was observed in a limited area around the Sylvian fissure adjacent to A1, whereas MMN was noted in wider areas, including the temporal, frontal, and parietal lobes. MMN was thus considered to be differentiated from N1 adaptation. The results suggest that MMN is not merely a product of the neural adaptation of N1 and instead represents higher-order processes in auditory deviance detection. These results will contribute to strengthening the foundation of future research in this field.

## Introduction

Auditory mismatch negativity (MMN) is an event-related potential (ERP) component induced by the oddball (OD) task, in which infrequent deviant tones intersperse a series of repetitive standard tones. MMN is obtained as a difference between the responses to the two different stimuli and shows a negative peak between 100 and 200 ms post-stimulus. MMN is induced even when the subject is unaware of stimuli, and is thus considered to reflect a certain process of cognitive function to detect a deviant stimulus pre-attentively (1). Previous studies on MMN were conducted with the aim of elucidating how perceived information, particularly auditory inputs, is processed and recognized in the brain (2–4). However, the generator and mechanism of auditory MMN remains unknown. The cortices adjacent to the primary auditory cortex in the temporal lobe are the most accepted candidates for containing the MMN generator, and previous studies have reported the involvement of the frontal lobe (5, 6).

The auditory ERP component called N1 is well known to be elicited by any auditory stimulus (7). This component is characterized by the first negative wave with a peak latency of approximately 100 ms poststimulus, which may originate in the lateral temporal plane (primary auditory cortex, or A1). Since N1 is evoked even by monotonous auditory stimuli, this component has been suggested to reflect a low-order auditory response of neural populations, mainly in the primary auditory cortex (8, 9). However, N1 is also elicited in the auditory OD task, which is used to obtain MMN. The possibility thus remains that MMN derives from a difference in N1 responsiveness to standard and deviant stimuli. Two conflicting hypotheses have been advocated, namely, the adaptation hypothesis and the deviance detection hypothesis.

The adaptation hypothesis assumes that N1 amplitude is reduced in the sequence of repetitive standard stimuli as a consequence of repetitive suppression and lateral inhibition in the poststimulus suppressive mechanism (10–13). Since this adaptation only occurs in a proportion of neural populations contributing to N1 generation in the primary auditory cortex, deviant stimuli may elicit the activation of non-adapted N1 neurons, yielding larger, and possibly enhanced N1 (14). According to this hypothesis, MMN is a product of the difference in N1 between adapted and non-adapted neural populations in the auditory cortices, elicited by standard and deviant stimuli, respectively. This hypothesis was supported by previous studies on animals including macaques (15), cats (16), and rats (17), as well as human studies combining functional magnetic resonance imaging (fMRI) with magneto-encephalography (MEG) (18, 19). On the other hand, MMN is generally accepted to reflect a higher-order function in auditory processing distinguished from the primary function represented by N1, and contributes to the prediction-based error detection system. The deviance detection hypothesis is supported by findings such as the localization discrepancy between MMN and N1 (20) and the observation of MMN even in the omission OD task, in which each deviant stimulus is replaced by a silent gap (21, 22).

We support the deviance detection hypothesis and assume that MMN is spatiotemporally distinguishable from N1 adaptation. In a human ECoG study, we have previously demonstrated that high gamma oscillation induced by the mismatch paradigm was widely distributed in the superior temporal gyrus, rather than within a limited area adjacent to the Sylvian fissure. Furthermore, mismatch response was shown to be attributable to deviance detection rather than adaptation (23). N1 adaptation did not account for the characteristics of the mismatch response. Based on the deviance detection hypothesis, the following four reasons appear to account for why MMN has not yet been clearly differentiated from N1 adaptation. First, differences in the distribution of MMN and N1 adaptation are not large enough to separate these components due to the limits of the signal-to-noise (S/N) ratio and the spatial resolution of conventional scalp electroencephalogram (EEG). Although the excellent spatiotemporal resolution of MEG has contributed to the current understanding of the sources of N1 and MMN (18, 19), application of the single dipole model shows limitations in localization, particularly for an extended source. Second, although a limited number of studies on MMN, referred to as mismatch response in some reports, have been performed using human intracranial recordings (24–26), all have focused solely on the distribution of MMN. No human intracranial study with the aim of differentiating MMN from N1 adaptation has been reported to date. Third, studies on MMN using rodents and non-human primates (11, 15–17, 27) were conducted based on electrophysiological data obtained around A1. Although these studies revealed that N1 adaptation occurred in A1, MMN may have been distributed outside the recording area and was thus considered negligible. Finally and most importantly, performing a spatial comparison between N1 adaptation and MMN as defined by their latencies seems difficult, as these values partially overlap in most cases. Since N1 adaptation occurs within the neural population that generates N1, the localization of N1 adaptation is assumed to be included in the N1 signal source. Different distributions of N1 and MMN mean that N1 adaptation is spatially separated from MMN.

To differentiate MMN and N1 adaptation, the present study aimed to separate MMN from N1 adaptation temporally, and then to separate MMN from N1 spatially using intracranial electrodes to cover the lateral surface of the brain widely and provide fine spatial resolution and a high S/N ratio.

## Materials and Methods

### Subjects

Three subjects (two males, one female) with refractory epilepsy participated in the present study. Subdural electrode placement was performed to identify epileptic foci and adjacent functional areas. Demographic characteristics of each subject are shown in **Table 1**. We confirmed that 1,000-Hz tones were detected at 30 dB in all subjects using audiometric testing.

**Table 1:** Subject demographics.

No.	Age/Sex	Duration of epilepsy (y)	Motor dominance	FIQ (in the range)	Electrode coverage	Epileptic foci	Etiology	AED
1	51/F	>30	Rt handed	70–80	Bil FTP	multiple	Post-herpes encephalitis	LEV, CBZ
2	27/M	<10	Rt handed	50–60	Lt FTP	Lt T	Not specified	LEV, CBZ
3	18/M	10–20	Rt handed	120–130	Bil FTP	Rt O	Rt O ganglioglioma	LEV, LTG, CBZ

FIQ, full-scale intelligence quotient; Lt, left; Rt, right; Bil, bilateral; F, frontal lobe; T, temporal lobe; P, parietal lobe; O, occipital lobe; med, medial; AED, anti-epileptic drug; LEV, levetiracetam; CBZ, carbamazepine; LTG, lamotrigine.

The present study was approved by the Ethics Review Board at the University of Tokyo Hospital. Written informed consent was obtained from all subjects and their family before participation in the present study.

### Task

We adopted the classical OD task, in which standard stimuli were presented with a high probability of 90% and deviant stimuli with a low probability of 10% in random order, yielding 1,200 trials. A 1,000-Hz tone with a length of 50 ms and a 1,200-Hz tone with a length of 50 ms were used as the standard and deviant stimuli, respectively.

As a control task, we used the many standard (MS) task. Ten different pitches of stimuli (every 100 Hz from 700 to 1,600 Hz) were used, including the same pitches as the standard and deviant tones in the OD task. Each stimulus was presented at the same probability of 10%. The MS task was assumed to avoid adaptation/enhancement, since each stimulus is assumed to recruit different frequency-specific neurons.

All stimuli used in the tasks were sinusoidal tones with an 80-dB sound pressure level (Multi Trigger System; Medical Try System, Tokyo, Japan). Stimulus onset asynchrony (SOA) was set to 500 ms. Tones were presented binaurally through inserted earphones while the subject was instructed to watch a silent video presented on a desktop monitor without paying attention to the auditory stimuli from the earphones.

### Data Acquisition

Subdural grid electrodes, consisting of silastic sheets with platinum electrodes (Unique Medical, Tokyo, Japan) were placed over the lateral surfaces of the frontal, temporal, and parietal lobes solely depending on the clinical purpose. Two types of grid electrodes were used in the present study: standard and higher-density electrodes with electrode diameters of 3 and 1.5 mm and inter-electrode (center-to-center) distances of 10 and 5 mm, respectively. Electrodes were placed for 2–4 weeks, during which time ECoG recordings for the present study were performed after obtaining clinically sufficient seizure information. No epileptic seizure events were identified in the 24 h before and after the recording.

ECoG data were recorded at a sampling rate of 2,000 Hz using a multi-channel EEG system (EEG 1200; Nihon Kohden, Tokyo, Japan). The band-pass filter for data acquisition was set at 0.09–600 Hz. A reference electrode was placed on the inner surface of the dura mater over the parietal lobe.

### Electrode Localization

Electrode locations were identified by post-implantation computed tomography registered to pre-implantation MRI based on the mutual information method using Dr.-View/Linux (Infocom, Tokyo, Japan). The three-dimensional brain surface with fused subdural electrodes was reconstructed using Real INTAGE (Cybernet Systems, Tokyo, Japan) for individual analysis.

### Data Analysis

All data were analyzed using custom script written in Matlab R2019b (MathWorks, Natick, MA) for each electrode. The band-pass filter between 2 and 30 Hz was applied to ECoG data. We chopped the filtered data from 250 ms prestimulus to 650 ms poststimulus for each stimulus into epochs of data. We defined data between 100 ms pre-stimulus and stimulus onset as the baseline. Each epoch was corrected for the baseline and was used in subsequent analyses.

In the MS task, all epochs were averaged, including those corresponding to the 10 different pitches of stimuli. We confirmed that the waveform and distribution of N1 evoked by all stimuli of the MS task did not differ from those with the 1,000-Hz tone only, and adopted all epochs of the MS task that included a higher number of trials and achieved higher S/N for the obtained N1. In the OD task, epochs of data were averaged separately for the standard and deviant stimuli. We then subtracted the average for the standard stimuli from that for deviant stimuli to obtain the subtraction waveform.

To validate the significance of N1 and MMN at each electrode, we defined N1 as the first negative peak observed at approximately 100 ms (from 80 to 120 ms) in the averaged waveform from the MS task, and MMN as the negative peak observed between 100 and 200 ms in the subtraction waveform from the OD task. We then performed a *t*-test for each electrode in every subject using corresponding epochs of data as follows. Each electrode was labeled as an N1 electrode when the potential of one of the time-points between 80 and 120 ms was significantly smaller than that at baseline. An MMN electrode was identified when the potential of one of the time-points between 100 and 200 ms in a deviant epoch was significantly smaller than that of the standard epochs. In each *t*-test, p < 0.05 was considered significant (corrected for multiple comparisons using false discovery rate [FDR] in terms of the numbers of channels and time points).

## Results

### Gross Observation of Waveforms at All Electrodes in the Three Subjects

In the averaged waveform from the MS task, the negative wave with a peak latency of approximately 100 ms poststimulus was observed at multiple electrodes in the superior temporal gyrus adjacent to the Sylvian fissure. In the subtraction waveform from the OD task, the negative wave with a peak latency between 100 and 200 ms was observed at multiple electrodes located in wide areas, including the temporal, parietal, and frontal lobes. These results in the MS and OD tasks were observed in common for all three subjects, supporting the tasks used in the present study as effectively evoking N1 and MMN.

### Temporal Comparison Between N1 Adaptation and MMN

**Figure 1:**
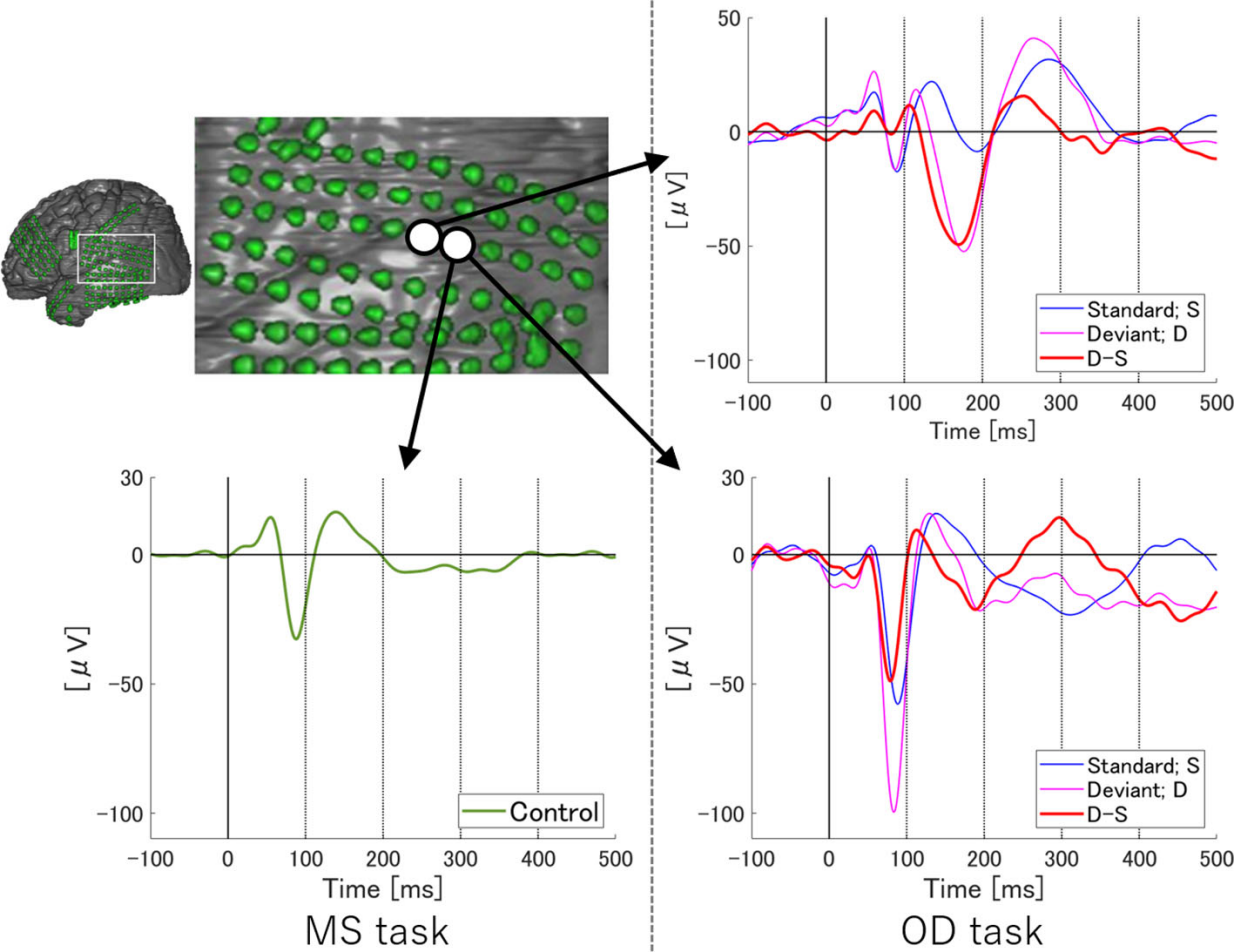
ERP at representative electrodes in Subject 1. Localization of representative electrodes and averaged waveforms at each electrode in Subject 1. Averaged waveforms in the many standards (MS) task, of standard stimuli in the oddball (OD) task, of deviant stimuli in the OD task, and subtraction waveform in the OD task are indicated by green, blue, pink, and red lines, respectively. At an electrode, we observe a negative wave with a peak latency of around 100 ms in the MS task (lower left). At the same electrode, similar negative waves are evoked by both standard and deviant stimuli in the OD task (lower right). Subtraction of these two waveforms yields a sharp negative wave with a peak latency of around 100 ms, as well as another negative wave between 100 and 200 ms. On the other hand, at the adjacent electrode, negative waves at around 100 ms evoked by standard and deviant stimuli in OD task are reduced (upper right). In the subtraction waveform, these two negative waves cancel each other out, yielding no obvious deflection at the N1 latency, but a negative wave between 100 and 200 ms.

**Figure 2:**
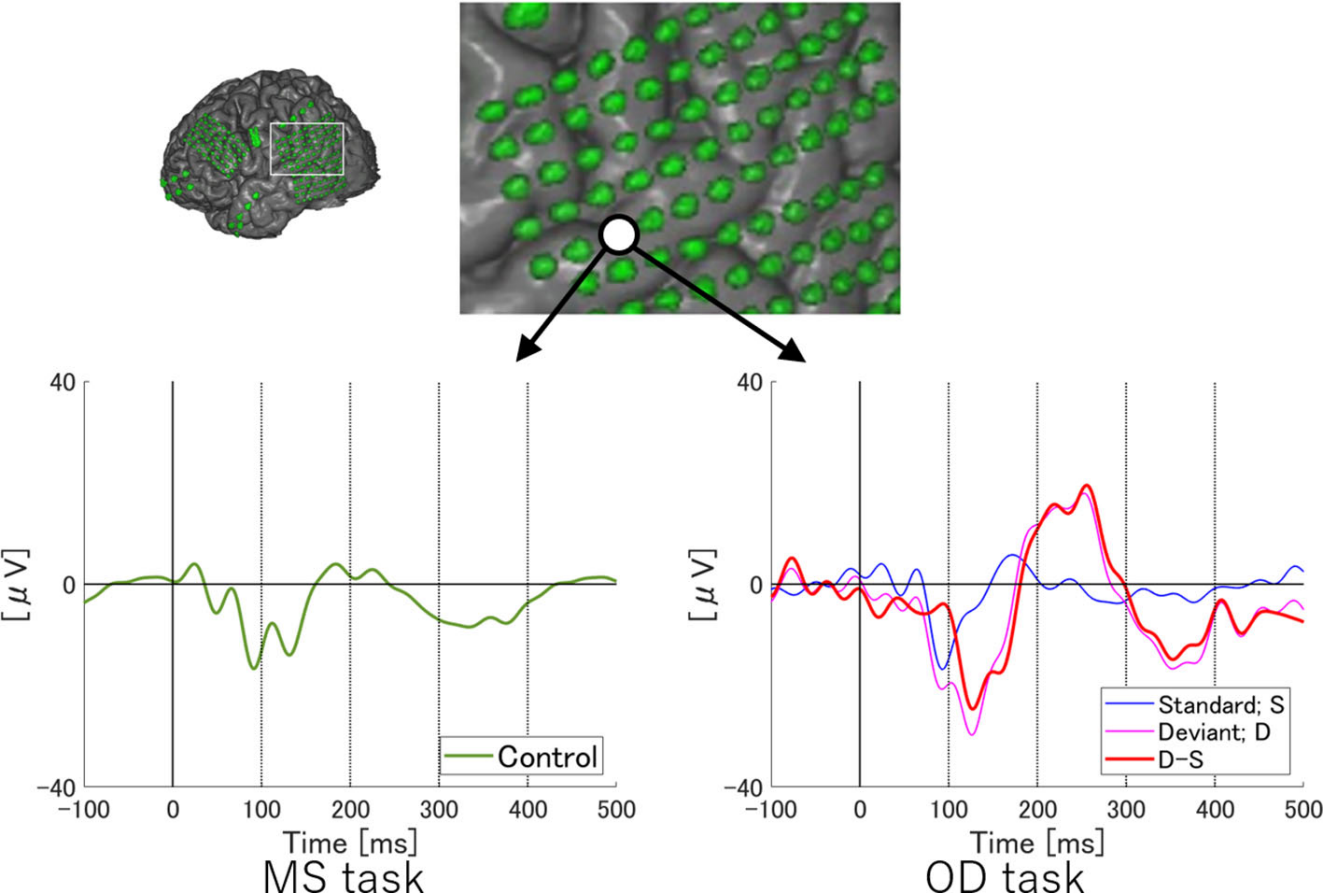
ERP at representative electrodes in Subject 2. Localization of a representative electrode and averaged waveforms at the electrode in Subject 2. Averaged waveforms in the many standards (MS) task, of standard stimuli in the oddball (OD) task, of deviant stimuli in the OD task, and the subtraction waveform in the OD task are indicated by green, blue, pink, and red lines, respectively. We observe a negative wave with a peak latency of around 100 ms in the MS task (lower left). At the same latency, negative waves are evoked by both standard and deviant stimuli in the OD task (lower right). In the subtraction waveform, these two negative waves cancel each other out, resulting in a slight negative potential. Subtraction of the waveforms yields obvious MMN at a relatively early latency. Separating the N1 adaptation and MMN in the subtraction waveform is no longer possible.

To examine temporal differences between N1 adaptation and MMN on single or adjacent electrodes at which both N1 and MMN were observed, we performed temporally detailed comparisons among the averaged waveforms from MS and OD tasks (**Figures 1** and **2**). At these electrodes, N1 was evoked by both standard and deviant stimuli in the OD task (upper right in **Figure 1**, right in **Figure 2**). Some of these evoked potentials canceled each other out in the subtraction waveform (upper right in **Figure 1**, right in **Figure 2**).

At one electrode in Subject 1, an outstanding negative wave was observed in the subtraction waveform at the same latency as N1 (lower right in **Figure 1**). The amplitude of N1 evoked by deviant stimuli was larger than that in the MS task, suggesting the involvement of N1 enhancement. At the same electrode, another negative wave between 100 and 200 ms poststimulus was also observed in the subtraction waveform, which corresponded to MMN based on a comparison with the obvious MMN recorded at the adjacent electrode. The finding that N1 adaptation and MMN were observed separately at different latencies at a single electrode suggests that N1 adaptation and MMN represent different responses *per se*. N1 adaptation at the adjacent electrode should be noted to have been noticeably reduced as a result of subtraction between N1s evoked by standard and deviant stimuli, leaving MMN alone. Evoked responses to standard and deviant stimuli thus varied markedly within 5 mm, and were only observed in a limited area at which the N1 and MMN electrodes coexisted.

On the other hand, at a single electrode in Subject 2, temporally separating N1 adaptation from MMN was difficult due to the following reasons. First, the negative deflection at the N1 latency in the subtraction waveform was unremarkable as a result of cancelation between responses to standard and deviant stimuli, although N1 itself was recognizable in MS task. Next, the peak latency of MMN observed in this subject was earlier than that in Subject 1. N1 adaptation thus appeared partly incorporated into MMN. Such incorporation of N1 adaptation into MMN was also observed in Subject 3.

As these results show, separating N1 adaptation and MMN in the same waveform was not necessarily possible. The following analysis therefore spatially compared MMN with N1 instead of N1 adaptation, with the underlying assumption that N1 adaptation is observed only at electrodes that show significant N1. Since the waveform in the MS task does not include MMN, N1 evoked by the MS task can be localized independently of MMN.

### Spatial Comparison Between N1 and MMN

We statistically defined the N1 electrode as an electrode showing a negative wave at approximately 100 ms in the MS task and the MMN electrode as that showing a negative peak between 100 and 200 ms poststimulus in the subtraction waveform from the OD task.

**Figure 3:**
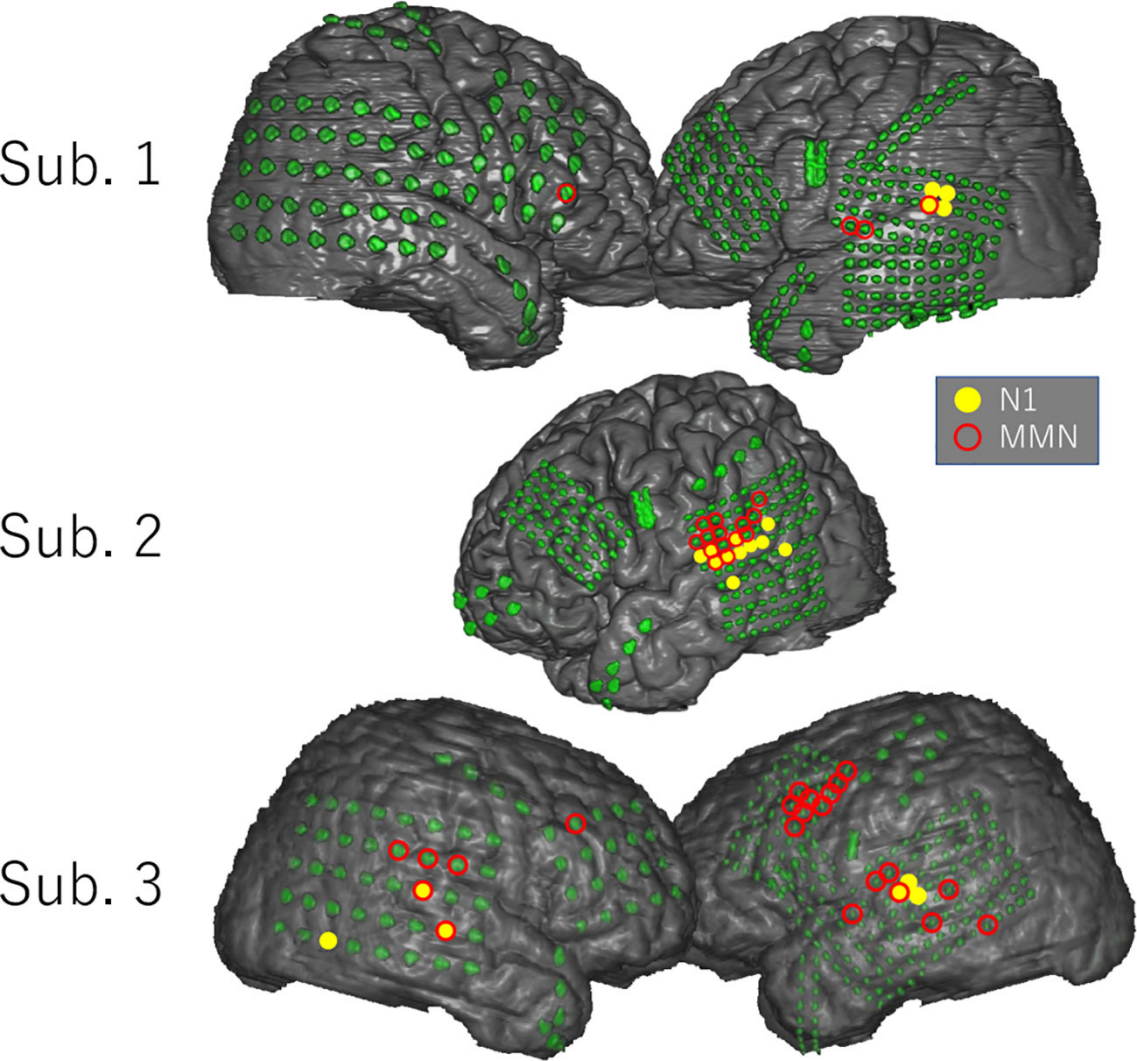
Distribution of N1 and MMN electrodes in each subject. N1 and MMN electrodes are statistically selected and indicated on the three-dimensional brain surface as yellow filled circles and red open circles, respectively. N1 electrodes except for one electrode in the inferior temporal gyrus of Subject 3 were located in the superior temporal gyrus near the superior temporal plane, whereas MMN electrodes were widely distributed in the superior and middle temporal gyrus, parietal lobe, and frontal lobe. The distribution pattern of MMN electrodes differed markedly among subjects.

**Figure 3** shows the distribution of the N1 and MMN electrodes of each subject. In every subject, N1 electrodes except for one electrode in the inferior temporal gyrus of Subject 3 were located in the superior temporal gyrus near the superior temporal plane, whereas MMN electrodes were widely observed in the superior and middle temporal gyrus, frontal lobe, and parietal lobe. Note that MMN response was poor in Subject 2 who had low FIQ. Although the localization of MMN electrodes overlapped with that of N1 electrodes in Subjects 2 and 3, the number of overlapping electrodes was lower than that of significant electrodes.

### Discussion

In the present study, human ECoG was performed during the MS and OD tasks in three patients with epilepsy for whom the lateral aspect of each hemisphere was widely covered by high-density grid electrodes. The results demonstrated that N1 adaptation/enhancement was temporally separate from MMN at a representative electrode. Detailed observation of the waveforms also revealed that N1 adaptation was not necessarily able to be temporally separated from MMN even using ECoG. We therefore compared the spatial distributions of N1 and MMN derived from different tasks based on the assumption that N1 adaptation/enhancement could occur only in the N1 population; this comparison successfully separated MMN from N1. The novel spatiotemporal differentiation of MMN and N1 adaptation revealed by this human ECoG study may provide important insights into auditory information processing.

### Temporal Separation Between N1 Adaptation and MMN

As shown in **Figure 1**, the N1 waveforms evoked by the standard and deviant stimuli of OD task canceled each other out at an electrode, whereas the difference between the two conditions formed a negative wave at the same latency as N1 at another electrode. These results in two adjacent electrodes indicate the involvement of N1 adaptation/enhancement in the OD task. However, these two electrodes also showed clear demarcation of MMN from N1 in the time course. The spatiotemporal pattern of ERP at these two electrodes, albeit under limited conditions, clearly demonstrates that MMN and N1 adaptation originate from different neural sources. Furthermore, the synchronicity of N1 latencies evoked by the standard and deviant stimuli suggested that the delayed N1 evoked by the deviant stimuli could not be the cause of MMN generation. Lower-density electrodes would have recorded these responses at a single electrode, resulting in a blurred picture of true ERP profiles and leading to difficulties differentiating between MMN and N1 adaptation, as has typically occurred in scalp EEG studies. This blurring effect from sampling errors is shown in **Figure 2**.

As depicted in the lower right of this negative waves at the same latency as N1 were noted just before MMN in the averaged waveforms from the OD task at a single electrode. Since N1 and MMN latencies were close at this electrode, the negative wave derived from the difference in N1 amplitude between standard and deviant stimuli was superimposed on the early phase of MMN, making the two components difficult to separate. The successful separation of MMN and N1 adaptation as independent ERP components in the same waveform appears to depend on individual differences in the spatiotemporal separation of the neural correlates of MMN and N1 and the spatial resolution of the recordings.

### Spatial Separation Between N1 and MMN

N1 adaptation and MMN show similar latencies. Defining the two components in the same waveform based on their latencies therefore results in a similar distribution of electrodes. This seems to be the primary reason behind the long-lasting controversy regarding differences between MMN and N1 adaptation. To address this issue, the present study compared the localizations of N1 and MMN. Use of the MS task as a control task enabled us to delineate pure N1 on the brain surface independently of MMN. Since N1 adaptation/enhancement occurs in the neural population that produces N1, the spatial separation of N1 and MMN indicates that the origin of N1 adaptation differs from that of MMN. Although a small number of MMN electrodes overlapped with N1 electrodes, the distribution of MMN was well-demarcated from that of N1. The adaptation hypothesis, which argues that MMN is only generated by neural populations recruited for N1, fails to account for the spatial dissociation between MMN and N1. These results were obtained through wide coverage of the lateral surface of the hemispheres by high-density ECoG electrodes, which was unique to the present study. Furthermore, the spatial comparison between N1 and MMN highlights the present study among the limited number of human ECoG studies that have focused solely on MMN.

In summary, MMN may be spatiotemporally differentiated from not only N1, but also the N1 adaptation; that is, the negative wave derived from the difference in N1 responsiveness to standard and deviant stimuli. The present study demonstrated the spatiotemporal differentiation of two components by performing spatial and temporal analyses separately. At the same time, the present study revealed that spatiotemporal differentiation of N1 adaptation and MMN required not only the use of high-density electrodes, but also proper comparison between N1 from the MS task and MMN from the OD task. The novel methodological combination implemented in the present study was what made these results possible which may provide further evidence for important and fundamental issues underpinning the framework of MMN studies.

### Limitation

In the present study, we showed MMN distributed in the larger areas than N1 using the OD and MS tasks. However, there are other MMN tasks using deviant stimuli such as duration deviance or omission deviance, which might have evoked different or even larger MMN responses in the lateral cortices (28). Moreover, oddball tasks with different oddball probabilities would have been helpful to identify MMN in a different way. In terms of a control task, a flip-flop task, in which the standard and deviant tones are flipped, would have been useful to eliminate the effect of the tone difference between the stimuli (29). Unfortunately, it was difficult to include the variety of the MMN tasks in the present study since the recording time for the MMN study was limited for clinical reasons. Although this issue does not reduce the validity of the present study essentially, combining the variety of these tasks is expected to enhance our knowledge and understanding of how and where MMN is generated.

## Conclusion

The present study demonstrated for the first time the spatiotemporal differentiation of MMN from N1 adaptation using human ECoG in combination with MS and OD tasks. Close observation of a representative electrode succeeded in temporally separating N1 adaptation from MMN. Spatial comparison between MMN and N1 obtained from the MS task instead of N1 adaptation revealed a separated distribution of N1 and MMN, suggesting that the origin of N1 adaptation differs from that of MMN. These results will contribute to strengthening the foundation of future research in this field.

## Data Availability Statement

The datasets presented in this article are not readily available because participants of this study did not agree for their data to be shared publicly. Requests to access the datasets should be directed to NK, nkunii-tky@umin.ac.jp.

## Ethics Statement

The studies involving human participants were reviewed and approved by the Ethics Review Board at the University of Tokyo Hospital. The patients/participants provided their written informed consent to participate in this study. Written informed consent was obtained from the individual(s) for the publication of any potentially identifiable images or data included in this article.

## Author Contributions

MeT, NK, MaT, KKi, and KKa conceptualized this research. MeT and YI performed data curation. MeT analyzed data. MeT, NK, MK, MaT, KKi, TU, and SS interpreted data. MeT and NK drafted the manuscript. MK, KKa, and NS supervised and revised the manuscript.

## Conflict of Interest

The authors declare that the research was conducted in the absence of any commercial or financial relationships that could be construed as a potential conflict of interest.
